# Toxicity of graphene-family nanoparticles: a general review of the origins and mechanisms

**DOI:** 10.1186/s12989-016-0168-y

**Published:** 2016-10-31

**Authors:** Lingling Ou, Bin Song, Huimin Liang, Jia Liu, Xiaoli Feng, Bin Deng, Ting Sun, Longquan Shao

**Affiliations:** 1 Nanfang Hospital, Southern Medical University, Guangzhou, 510515 China; 2 The First Affiliated Hospital of Jinan University, Guangzhou, China; 3 The General Hospital of People’s Liberation Army, Beijing, China

**Keywords:** Graphene-family nanomaterials, Toxicity, Toxicokinetics, Mechanisms, Physicochemical properties, Future prospects

## Abstract

Due to their unique physicochemical properties, graphene-family nanomaterials (GFNs) are widely used in many fields, especially in biomedical applications. Currently, many studies have investigated the biocompatibility and toxicity of GFNs in vivo and in intro. Generally, GFNs may exert different degrees of toxicity in animals or cell models by following with different administration routes and penetrating through physiological barriers, subsequently being distributed in tissues or located in cells, eventually being excreted out of the bodies. This review collects studies on the toxic effects of GFNs in several organs and cell models. We also point out that various factors determine the toxicity of GFNs including the lateral size, surface structure, functionalization, charge, impurities, aggregations, and corona effect ect. In addition, several typical mechanisms underlying GFN toxicity have been revealed, for instance, physical destruction, oxidative stress, DNA damage, inflammatory response, apoptosis, autophagy, and necrosis. In these mechanisms, (toll-like receptors-) TLR-, transforming growth factor β- (TGF-β-) and tumor necrosis factor-alpha (TNF-α) dependent-pathways are involved in the signalling pathway network, and oxidative stress plays a crucial role in these pathways. In this review, we summarize the available information on regulating factors and the mechanisms of GFNs toxicity, and propose some challenges and suggestions for further investigations of GFNs, with the aim of completing the toxicology mechanisms, and providing suggestions to improve the biological safety of GFNs and facilitate their wide application.

## Background

Graphene, which is isolated from crystalline graphite, is a flat monolayer composed of single-atom-thick, two-dimensional sheets of a hexagonally arranged honeycomb lattice [1]. Because of its unique structural, specific surface area and mechanical characteristics, the functions and applications of graphene have gained considerable attention since the discovery of the material in 2004 [2, 3]. Graphene and its derivatives include monolayer graphene, few-layer graphene (FLG), graphene oxide (GO), reduced graphene oxide (rGO), graphene nanosheets (GNS), and graphene nanoribbons, etc. [4–7]. GO is one of the most vital chemical graphene derivatives of the graphene-family nanomaterials (GFNs), which attracts increasing attention for its potential biomedical applications. Graphene-based materials usually have sizes ranging from several to hundreds of nanometer and are 1-10 nm thick [8, 9], which is also the definition of ‘nanoparticles’ or ‘nanomaterials’. Due to their exceptional physical and chemical properties, graphene materials have been widely used in various fields, including energy storage; nanoelectronic devices; batteries [10–12]; and biomedical applications, such as antibacterials [13, 14], biosensors [15–18], cell imaging [19, 20], drug delivery [8, 21, 22], and tissue engineering [23–25].

Along with the application and production of GFNs increasing, the risk of unintentional occupational or environmental exposure to GFNs is increasing [26]. And recently, there are some investigation on GFNs exposure in occupational settings and published data showed that the occupational exposure of GFNs had potential toxicity to the workers and researchers [27–29]. GFNs can be delivered into bodies by intratracheal instillation [30], oral administration [31], intravenous injection [32], intraperitoneal injection [33] and subcutaneous injection [34]. GFNs can induce acute and chronic injuries in tissues by penetrating through the blood-air barrier, blood-testis barrier, blood-brain barrier, and blood-placenta barrier etc. and accumulating in the lung, liver, and spleen etc. For example, some graphene nanomaterials aerosols can be inhaled and substantial deposition in the respiratory tract, and they can easily penetrate through the tracheobronchial airways and then transit down to the lower lung airways, resulting in the subsequent formation of granulomas, lung fibrosis and adverse health effects to exposed persons [2, 29]. Several reviews have outlined the unique properties [35, 36] and summarized the latest potential biological applications of GFNs for drug delivery, gene delivery, biosensors, tissue engineering, and neurosurgery [37–39]; assessed the biocompatibility of GFNs in cells (bacterial, mammalian and plant) [7, 40, 41] and animals (mice and zebrafish) [42]; collected information on the influence of GFNs in the soil and water environments [43]. Although these reviews discussed the related safety profiles and nanotoxicology of GFNs, the specific conclusions and detailed mechanisms of toxicity were insufficient, and the mechanisms of toxicity were not summarized completely. The toxicological mechanisms of GFNs demonstrated in recent studies mainly contain inflammatory response, DNA damage, apoptosis, autophagy and necrosis etc., and those mechanisms can be collected to further explore the complex signalling pathways network regulating the toxicity of GFNs. It needs to point out that there are several factors which largely influence the toxicity of GFNs, such as the concentration, lateral dimension, surface structure and functionalization etc. Herein, this review presents a comprehensive summary of the available information on the mechanisms and regulating factors of GFNs toxicity in vitro and in vivo via different experimental methods, with the goals of providing suggestions for further studies of GFNs and completing the toxicology mechanisms to improve the biological safety of GFNs and facilitate their wide application.

## Toxicity of GFNs (in vivo and in vitro)

GFNs penetrate through the physiological barriers or cellular structures by different exposure ways or administration routes and entry the body or cells, eventually resulting in toxicity in vivo and in vitro. The varying administration routes and entry paths, different tissue distribution and excretion, even the various cell uptake patterns and locations, may determine the degree of the toxicity of GFNs [44–46]. So to make them clear may be helpful to better understand the laws of the occurrence and development of GFNs toxicity.

### Administration route

The common administration routes in animal models include airway exposure (intranasal insufflation, intratracheal instillation, and inhalation), oral administration, intravenous injection, intraperitoneal injection and subcutaneous injection. The major exposure route for GFNs in the working environment is airway exposure, thus inhalation and intratracheal instillation are used mostly in mice to simulate human exposure to GFNs. Though the inhalation method provides the most realistic simulation to real life exposure, instillation is more effective and time-saving method, and GFNs was found that causing longer inflammation period using instillation (intratracheal instillation, intrapleural installation and pharyngeal aspiration) than inhalation [24, 30, 47, 48]. GFNs were investigated to deposit in the lungs and accumulate to a high level, which retained for more than 3 months in the lungs with slow clearing after intratracheal instillation [49]. Intravenous injection is also widely used to assess the toxicity of graphene nanomaterials, and graphene circulates through the body of mice in 30 min, accumulating at a working concentration in the liver and bladder [32, 50–52]. However, GO derivatives had rather finite intestinal adsorption and were rapidly excreted in adult mice via oral administration [31, 53]. Nano-sized GO (350 nm) caused less mononuclear cells to infiltrate subcutaneous adipose tissue after subcutaneous injection in the neck region compared to micron-sized GO (2 μm) [34]. GO agglomerated near the injection site after intraperitoneal injection, and numerous smaller aggregates settled in the proximity of the liver and spleen serosa [31, 33]. Experiments on skin contact with or skin permeation of GFNs were not found in the papers reviewed here, and there is insufficient evidence available to conclude that graphene can penetrate intact skin or skin lesions. The route of nasal drops, which has been widely used to test the neurotoxicity or brain injury potential of other nanomaterials, was not mentioned in the papers reviewed here.

### GFNs entry paths

GFNs reach various locations through blood circulation or biological barriers after entering the body, which results in varying degrees of retention in different organs. Due to their nanosize, GFNs can reach deeper organs by passing through the normal physiological barriers, such as the blood-air barrier, blood-testis barrier, blood-brain barrier and blood-placental barrier.

#### Blood-air barrier

The lungs are a potential entrance for graphene nanoparticles into the human body through airway. The inhaled GO nanosheets can destroy the ultrastructure and biophysical properties of pulmonary surfactant (PS) film, which is the first line of host defense, and emerge their potential toxicity [54]. The agglomerated or dispersed particles deposit on the inner alveolar surface within the alveoli and then be engulfed by alveolar macrophages (AMs) [55]. Clearance in the lungs is facilitated by the mucociliary escalator, AMs, or epithelial layer [56–58]. However, some small, inhaled nanoparticles infiltrate the intact lung epithelial barrier and can then transiently enter the alveolar epithelium or the interstitium [59, 60]. Intratracheally instilled graphene can redistribute to the liver and spleen by passing through the air-blood barrier [61]. The study of blood-air barrier may draw an intensive attention, since the researchers and workers occupational exposure of GFNs usually through inhalation. To make clear how the blood-air barrier plays a role in the toxicity of GFNs may become a research hot topic.

#### Blood-brain barrier

The intricate arrangement of the blood-brain barrier, consisting of numbers of membrane receptors and highly selective carriers, only exerts subtle influence on blood circulation and the brain microenvironment compared to the peripheral vascular endothelium [62]. The research on the mechanism of blood-brain barrier had made some progress involved in diseases and nanotoxicity. Matrix-assisted laser desorption/ionization (MALDI) mass spectrometry imaging (MSI) revealed that rGO, with an average diameter of 342 ± 23.5 nm, permeated through the paracellular pathway into the inter-endothelial cleft in a time-dependent manner by decreasing the blood-brain barrier paracellular tightness [63]. In addition, graphene quantum dots (GQDs), with a small size of less than 100 nm, can cross through the blood-brain barrier [64]. Studies on how graphene materials pass through the blood-brain barrier and cause neurotoxicity are very rare, and more data are needed to draw a conclusion.

#### Blood-testis barrier

The blood-testis and blood-epididymis barriers are well known for being some of the tightest blood-tissue barriers in the mammalian body [65]. GO particles with diameters of 54.9 ± 23.1 nm had difficulty penetrating the blood-testis and blood-epididymis barriers after intra-abdominal injection, and the sperm quality of the mice was not obviously affected even at 300 mg/kg dosage [66].

#### Blood-placenta barrier

The placental barrier is indispensable in maintaining pregnancy, as it mediates the exchange of nutrients and metabolic waste products, exerts vital metabolic functions and secretes hormones [67]. A recent review suggested that the placenta does not provide a tight barrier against the transfer of nanoparticles to foetuses, specifically against the distribution of carbonaceous nanoparticles to and in the foetus [42]. It was suggested that rGO and gold particles (diameter of 13 nm) are barely present or are absent in the placenta and foetus in late gestation after intravenous injection [44, 68]. However, other reports showed that transplacental transfer does occur in late gestational stages [69, 70]. Much attention had been paid to the developmental toxicity of nanomaterials, and reports showed that many nanoparticles did cross the placental barrier and strongly influenced the development of embryos [71–75]. But studies of the exposure to graphene materials through the placenta barrier are deficient, and how these particles transfer to embryos should be evaluated in detail in the future.

These four barriers were the most frequently mentioned barriers in the literature, and other barriers have not been evaluated in recent studies, such as skin barriers, which have not been mentioned in any of the hundreds of GFNs toxicity studies searched. Moreover, the mechanism by which GFNs pass through these barriers is not well understood, and more systematic investigations are urgently needed.

### Distribution and excretion of GFNs in tissue

The absorption, distribution, and excretion of graphene nanoparticles may be affected by various factors including the administration routes, physicochemical properties, particle agglomeration and surface coating of GFNs.

The different administration routes influence the distribution of GFNs, for example, intratracheally instilled FLG passing through the air-blood barrier mainly accumulated and was retained in the lungs, with 47 % remaining after 4 weeks [61]. Intravenously administered GO entered the body through blood circulation and was highly retained in the lung, liver, spleen and bone marrow, and inflammatory cell infiltration, granuloma formation and pulmonary edema were observed in the lungs of mice after intravenous injection of 10 mg kg/body weight GO [49]. Similarly, high accumulation of PEGylated GO derivatives was observed in the reticuloendothelial (RES) system including liver and spleen after intraperitoneal injection. In contrast, GO-PEG and FLG did not show detectable gastrointestinal tract absorption or tissue uptake via oral administration [31].

The different properties of GFNs, such as their size, dose and functional groups, always lead to inconsistent results in the distribution profiles of graphene. For instance, Zhang et al. found that GO was mainly entrapped in mouse lungs [49]; however, Li et al. observed that GO accumulated in mouse liver [76]. Notably, small GO sheets, with diameters of 10–30 nm, were mainly distributed in the liver and spleen, whereas larger GO sheets (10–800 nm) mainly accumulated in the lungs [49, 52, 77]. If the size of GO is larger than the size of the vessels, GO usually becomes stuck in the arteries and capillaries in the proximity of the injection site. The accumulation of GO in the lungs was shown to increase with an increase in the injected dose and size, but that in the liver significantly decreased [78]. Coating biocompatible polymers onto GO also affects the biodistribution, for instance, the intravenous injection of GO-PEG and GO-dextran (GO-DEX) accumulate in the reticuloendothelial system (RES), including the liver and spleen, without short-term toxicity [31, 79]. Moreover, the charge of plasma proteins and adsorption of GO by plasma proteins also affects the biodistribution [34].

The excretion and clearance of GFNs vary in different organs. In the lungs, observations indicated that NGO is drawn into and cleared by AMs, which might be eliminated from the sputum through mucociliary clearance or other ways [57], and 46.2 % of the intratracheally instilled FLG was excreted through the faeces 28 d after exposure [61]. In the liver, nanoparticles can be eliminated thorough the hepato-biliary pathway following the biliary duct into the duodenum [80]. In addition, PEGylated GNS that mainly accumulates in the liver and spleen can be gradually cleared, likely by both renal and faecal excretion. As recently reviewed, GO sheets larger than 200 nm are trapped by splenic physical filtration, but small sizes (approximately 8 nm) can penetrate the renal tubules into the urine and be rapidly removed without obvious toxicity [81]. The excretion paths of GFNs have not yet been clearly explained, but renal and faecal routes appear to be the main elimination routes for graphene.

Recently, the distribution and excretion/toxicity strategy has become an important part of nano-toxicological studies. To date, several controversial results regarding the distribution and excretion of graphene in vivo have been reported in several papers, and a systematic evaluation of the toxicokinetics of GFNs is still needed. The metabolism and excretion of nanomaterials are long-period processes, however, the recent studies of GFNs had been limited to short-term toxicological assessments, and the long-term accumulation and toxicity of GFNs on different tissues remain unknown. Therefore, long-term studies on the deposition and excretion of GFNs need to be performed using different cells and animals to ensure the materials’ biosafety before utilization in human biomedical applications.

### Uptake and location of GFNs in cells

The uptake and location of GFNs have also been observed to exert different effects in different cell lines. Graphene is taken up into cells via various routes [82, 83]. Basically, the physicochemical parameters such as the size, shape, coating, charge, hydrodynamic diameter, isoelectric point, and pH gradient are important to allow GO to pass through the cell membrane [84]. As stated previously, nanoparticles with diameters <100 nm can enter cells, and those with diameters <40 nm can enter the nucleus [85]. For example, GQDs possibly penetrate cell membranes directly, rather than through energy-dependent pathways [86, 87]. Larger protein-coated graphene oxide nanoparticles (PCGO) (~1 μm) enter cells mainly through phagocytosis, and smaller PCGO nanoparticles (~500 nm) enter cells primarily through clathrin-mediated endocytosis [88]. GO sheets could adhere and wrap around the cell membrane, insert in the lipid bilayer or be internalized into the cell as a consequence of interactions with cells [89]. Similarly, PEGylated reduced graphene oxide (PrGO) and rGO were shown to adhere onto the lipid bilayer cell membrane prominently due to the interaction of hydrophobic, unmodified graphitic domains with the cell membrane [90, 91]. Consequently, it was suggested that prolonged exposure to or a high concentration of graphene induces physical or biological damage to the cell membrane, along with destabilization of actin filaments and the cytoskeleton [92].

**Fig. 1:**
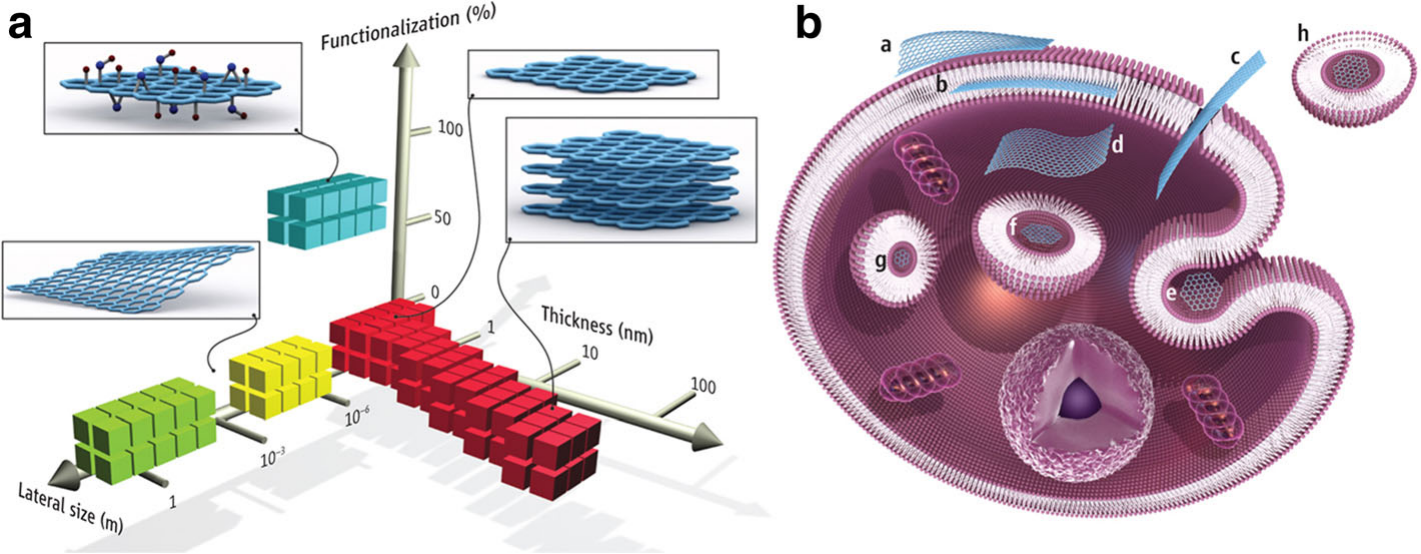
Graphene materials and their biological interactions. (**A**) A parameter space for the most widely used graphene materials can be described by the dimensions and surface functionalization of the material, the latter defined as the percentage of the carbon atoms in sp3 hybridization. Green squares represent epitaxially grown graphene; *yellow*, mechanically exfoliated graphene; *red*, chemically exfoliated graphene; *blue*, graphene oxide. Note that a number of other graphene-related materials (such as graphene quantum dots and graphene nanoribbons) are also being used in experiments. (**B**) Possible interactions between graphene-related materials with cells (the graphene flakes are not to scale). (*a*) Adhesion onto the outer surface of the cell membrane. (*b*) Incorporation in between the monolayers of the plasma membrane lipid bilayer. (*c*) Translocation of membrane. (*d*) Cytoplasmic internalization. (*e*) Clathrin-mediated endocytosis. (*f*) Endosomal or phagosomal internalization. (*g*) Lysosomal or other perinuclear compartment localization. (*h*) Exosomal localization. The biological outcomes from such interactions can be considered to be either adverse or beneficial, depending on the context of the particular biomedical application. Different graphene-related materials will have different preferential mechanisms of interaction with cells and tissues that largely await discovery. [90] Copyright (2014), with permission from American Association for Advancement of Science

Current data demonstrates that GO sheets interact with the plasma membrane and are phagocytosed by macrophages. Three major receptors on macrophages take part in the phagocytosis of GNS: the Fcg receptor (FcgR), mannose receptor (MR), and complement receptor (CR). Furthermore, FcgR is a key receptor in the mediated phagocytic pathway [90, 93, 94]. The protein corona of GO promotes the recognition by macrophage receptors, especially the IgG contained within the protein corona. Macrophages were observed to undergo prodigious morphological changes upon contact with GO [34]. After internalization, graphene accumulated in the cell cytoplasm, perinuclear space, and nucleus, which induced cytotoxicity in murine macrophages by increasing intracellular ROS through depletion of the mitochondrial membrane potential and by triggering apoptosis through activation of the mitochondrial pathway [83]. The possible interactions and accumulation sites of GFNs are summarized in Fig. 1.

### Toxicity of GFNs in organs

The toxicity and biocompatibility of GFNs has been observed and assessed through theoretical and animal model studies. At present, there are a mass of data demonstrating the toxicity of GFNs in different organs or systems in animals, so that it is hard to list all the data in this review. Thus we summarized a certain number literature and chose some in vivo toxicological studies of GFNs listed in Table 1.

**Table 1:** Toxicity of GFNs in organs

Graphene family nanomaterials	Physiochemial properties and functionalization	Animals	Dose and time incubation	Effects	Reference
Nanoscale graphene oxide (NGO)	No information	C57BL/6 mice	0, 1, 5, 10 mg/kg, intratracheal instillation 0 h, 24 h, 48 h, 72 h and 1 week	Result in acute lung injury (ALI) and chronic pulmonary fibrosis	[30]
Few layer graphene (FLG)	No information	ICR mice	0.1, or 1 mg/mL, oral gavage or intratracheal instillation 3 or 28 days	Intratracheally instilled FLG resulted in acute lung injury and pulmonary edema, FLG didn’t show detectable absorption through the gastrointestinal tract by oral gavage.	[61]
Graphene platelets (GPs)	No information	Mice	inhalation exposure, 1 day-6 weeks	GP caused acute inflammation in lung at 1 day, and alleviated inflammation in lung after 6 weeks	[48]
Graphene nanoplatelets (GPs)	Thickness of 10 nm Size of 5–30 μm	Female C57BL/6 strain mice	50 μg per mouse, pharyngeal aspiration or intrapleural installation, 24 h- 7 days	Large GP were inflammogenic in both the lung and the pleural space	[24]
GO	Thickness of 0.93 nm Size of 150–250 nm	Sprague-Dawley rats	0.5 or 4 mg/m3, inhalation exposure, single 6 h	The single inhalation exposure to GO induce minimal toxic responses in rat lungs	[235]
GO	Thickness of 0.9 nm size of l-GO: 1–5 μm size of s-GO:100–500 nm	Male ICR mice	1.0 mg/kg, intravenous injected, 24 h	Accumulated mainly in the liver and lungs	[78]
GO	Thickness of < 4 nm size of l-GO:237.9 ± 79.3 nm; size of s-GO: 54.9 ± 23.1 nm	Male and female ICR-strain mice	24 mg/kg, tail vein injected, 5 days	Didn’t effect pup numbers, sex ratio, weights, pup survival rates or pup growth, low toxicity for male reproduction	[66]
GO	Thickness of ~1.0 nm sizes of 10–800 nm	Kun Ming mice	1,10 mg/ kg, intravenous injection 14 days	Led to high accumulation, long-time retention, pulmonary edema and granuloma formation	[49]
NGO-PEG	Thickness of 1 nm size of 10–800 nm	Male Kunming mice	5 mg/kg, tail intravenous injection 10 min-24 h	NGO-PEG alleviated acute tissue injuries, decreased the early weight loss	[81]
GO GO-PEG RGO-PEG nRGO-PEG	Thickness of 0.94,1.22, 4.43 and 5.66 nm, size of 450, 25, 50 and 27 nm	Balb/c mice	4 mg/kg, intraperitoneal injection 1, 7 and 30 days	Accumulated in the reticuloendothelial (RES) system including liver and spleen over a long time	[31]
GO Graphene quantum dots (GQD)	Thickness of GO, GQD: 0.5–1 nm sizes of GO, GQD: 3–5 nm	Balb/c mice	20 mg/kg intravenous injection or intraperitoneal injection 14 days	GO appeared toxic and caused death GQD revealed no accumulation in organs and caused low cytotoxicity	[176]
Purified graphene oxide (pGO)	Thickness of 1–2 nm, lateral dimension of 100–500 nm	Female C57Bl/6 mice	50 μg/animal, intraperitoneal injection 24 h, 7 days,	Induced moderate inflammation and granuloma formation following	[99]
GO	Thickness of 3.9 and 4.05 nm, size of 350 nm and 2 μm	C57BL/6 male mice	Series concentrations, subcutaneous injection21 days	The micro-size of GO induced much stronger inflammation responses than the nanosized GO	[34]
GO	Size of 1110 to 16 200 nm	C57BL/6 J mice	2 or 20 mg/kg, subcutaneous and intraperitoneal injection	Both GO and a reduction of GO result in immune cell infiltration, uptake, and clearance.	[84]
RGO-iron oxide nanoparticles (rGO-IONP)	Thickness of ˂10 nm Size of 15.0 ± 2.0 nm	Female Balb/c mice	400 μg, subcutaneous injection,	RGO–IONP can effectively inactivate multiple-drug-resistant bacteria in subcutaneous abscesses	[236]
GO GO-PEG	Thickness of 0.94, 1.22, 4.43 and 5.66 nm, size of 450, 25, 50 and 27 nm	Female balb/c mice	100 mg/kg, Oral administration; 50 mg/kg, intraperitoneal injection, 1, 7 and 30 days	No obvious tissue uptake via oral administration, indicating the rather limited intestinal adsorption of those nanomaterials	[237]
RGO	sizes of small rGO: 87.97 ± 30.83, sizes of large rGO:472.08 ± 249.17 nm	Male C57black/6 mice	60 mg/kg, oral gavage, 5 days	RGO affected general locomotor activity, balance, and neuromuscular coordination, but showed little change in exploratory, anxiety-like, or learning and memory behaviors.	[31]

#### Toxicity in internal organs

GO can result in acute inflammation response and chronic injury by interfering with the normal physiological functions of important organs [32, 81]. Oral gavage experiments did not show detectable absorption of GO through the gastrointestinal tract [95]. Interesting, a low dose of GO caused serious damage to the gastrointestinal tract after maternal mice drank a GO suspension rather than a high-dose of GO because a low dose of GO without agglomeration can easily attach to the gastrointestinal surface and cause destruction through its abundant sharp edges [53]. GFNs caused inflammation and remained in the lung on day 90 after a single intratracheal instillation, and even translocated to lung lymph nodes by a nose-only inhalation [96, 97]. A high dose of GO that forms aggregations can block pulmonary blood vessels and result in dyspnea [50, 98], and platelet thrombi were observed at high concentrations of 1 and 2 mg/kg body weight via intravenous injection [89]. GO reportedly disrupted the alveolar-capillary barrier, allowing inflammatory cells to infiltrate into the lungs and stimulate the release of pro-inflammatory cytokines [99]. Fibrosis and inflammation could be verified by the increased levels of the protein markers collagen1, Gr1, CD68 and CD11b in the lungs. The use of Tween 80 to disperse FLG or a pluronic surfactant to disperse graphene was suggested to reduce the likelihood of lung fibrosis formation in cells or mice, whereas lung fibrosis was observed when graphene was suspended with bovine serum albumin (BSA) [100]. In addition, radioactive isotopes can be delivered into the lungs, accompanied by a depth distribution of ^125^I-NGO in the lungs, and the isotopes might deposit there and result in mutations and cancers [30]. However, recent publications claimed no obvious pathological changes in mice exposed to low dosages of GO and functionalized graphene by intravenous injection, including aminated GO (GO-NH2), poly(acrylamide)-functionalized GO (GO-PAM), poly(acrylic acid)-functionalized GO (GO-PAA) and GO-PEG; only GO-PEG and GO-PAA induced less toxicity than pristine GO in vivo [31, 79, 89]. So the functional groups of GFNs and the working concentration or aggregate state largely influence the toxicity of GFNs. Recently, the ways to modify the functional group of GFNs, decrease the working concentration or change the aggregate condition are usually used to decrease the toxicity of GFNs.

#### Toxicity in the central nervous system

Graphene has largely benefited neurosurgery with the application of drug/gene delivery for brain tumour treatment, intracranial and spinal biocompatible devices, biosensing and bioimaging techniques. Studies regarding the potentialities or risks of graphene in the brain have emerged. In the chicken embryo model, pristine graphene flakes decreased the ribonucleic acid level and the rate of deoxyribonucleic acid synthesis, leading to harmful effects on brain tissue development and the atypical ultrastructure was observed in the brain [101]. The recent researches of GFNs in the central nervous system are mostly involved in the application rather than the toxicity. The data of the toxic study on GFNs is underway.

#### Toxicity in reproduction and development system

Pristine graphene reduced the vascularization of the heart and the density of branched vessels after injection into fertilized chicken eggs followed by incubation for 19 d [101]. GO and rGO damage zebrafish embryos by influencing the embryo hatching rate and body length in a concentration-dependent manner. Although no obvious malformation or mortality was observed in exposed zebrafish embryos [102], GO adhered to and was wrapped in the chorion of the zebrafish embryos, causing remarkable hypoxia and hatching delay. GO aggregates were retained in many organelles, such as the eyes, heart, yolk sac, and tail of the embryos, and apoptosis and reactive oxygen species (ROS) generation were observed in these regions [103].

The GFNs exert different toxicological effects on male or female reproductive system. Data showed that GO exerted very low or nearly no toxic effects on male reproduction even at a high dose via intra-abdominal injection [66]. Additionally, rGO did not change the serum estrogen levels of non-pregnant female mice. The condition is different in the female mouse: mouse dams could give birth to healthy offspring after rGO injection before mating or during early gestation, and only a few abnormal foetuses were present among the rGO-injected dam litters. However, the pregnant mice had abortions at all dose, and most pregnant mice died when the high dose of rGO was injected during late gestation [44]. Notably, the development of offspring in the high dosage group was delayed during the lactation period. The high dose of GO decreased the maternal mice’s water consumption by oral exposure, which reduced milk production and thus postponed the growth of offspring [53]. Though the findings indicate that GFNs are potentially harmful to development, but data on reproductive and developmental toxicity are still deficient. Studies of the influence of GFNs on male and female reproduction and development are still required to elucidate the underlying toxicity mechanism.

#### Influence of haemocompatibility

GO release into the blood is ineluctable. The haemocompatibility of GO was found to be dependent on the functional coating and the exposure conditions. GO with submicron size resulted in the greatest haemolytic activity, while aggregated graphene induced the lowest haemolytic reaction. Pristine graphene and GO demonstrated haemolytic effect up to 75 μg/mL [104]. GO-polyethylenimine (GO-PEI) exhibited notable toxicity by binding to HSA, even at 1.6 μg/mL [105]. Carboxylated graphene oxide (GO-COOH) showed significant cytotoxicity toward T lymphocytes at concentrations above 50 μg/mL and had good biocompatibility below 25 μg/mL, whereas GO-chitosan nearly inhibited haemolytic activity [106]. Until now, the corresponding risk of haemocompatibility has remained largely unknown.

In conclusion, the lung injury induced by GFNs has been studied in several studies, the results of which have demonstrated inflammatory cell infiltration, pulmonary edema and granuloma formation in the lungs. However, only a few specific studies have evaluated in other organs, such as the liver, spleen, and kidney, and the injury symptoms, damage index and level of damage to these internal organs were not fully investigated. Moreover, studies on the neurotoxicity of GFNs are quite rare; no data has revealed which nerves or brain areas experience damage, nor have the related behavioural manifestations been studied. The developmental toxicity of GFNs may induce structural abnormalities, growth retardation, behavioural and functional abnormalities, and even death. A study on the reproductive and developmental toxicity of GFNs will be extremely significant and gain extensive attention in the future. Almost all the GFNs toxicity studies were short-period experiments, and no studies have investigated long-term chronic toxic injury. However, based on studies of other nanomaterials toxicity, long-term GFNs exposure may be an important factor harming health [107–109]. Therefore, the long-term study of GFNs is necessary.

### Toxicity of GFNs in cell models

The cytotoxicity of GFNs in vitro has been verified in various cells to change the cell viability and morphology, destroy the membrane integrity, and induce DNA damage [110–112]. GO or rGO decrease cell adhesion; induce cell apoptosis; and enter lysosomes, mitochondria, cell nuclei, and endoplasm [113]. GQDs entered cells and induced DNA damage by the increased expression of p53, Rad 51, and OGG1 proteins in NIH-3 T3 cells [87]. However, GQDs did not pose significant toxicity to human breast cancer cell lines (at a dose of 50 μg/mL) or human neural stem cells (at a dose of 250 μg/mL) [114, 115]. GO derivatives dramatically decreased the expression of differential genes that are responsible for the structure and function of the cell membrane, such as regulation of the actin cytoskeleton, focal adhesion and endocytosis [89]. In rat pheochromocytoma cells (PC12 cells), graphene and rGO caused cytotoxic effects and mitochondrial injury, such as the release of lactate dehydrogenase (LDH), an increase in the activation of caspase-3, and the generation of ROS [82, 116].

Graphene can increase cell viability [117] or cause cell death [118] depending on the cell line, type of graphene material and the doseage. GO cytotoxicity was observed in human fibroblasts and lung epithelial cells at concentrations above 20 μg/mL after 24 h, but minimal toxicity was found in A549 cells at concentrations higher than 50 μg/mL [119]. The biological responses induced by GO such as ROS, malondialdehyde (MDA), and LDH increased, whereas superoxide dismutase (SOD) decreased dose-dependently in HeLa cells [120]. However, GO-molecular beacon (GO-MB) showed low cytotoxicity even at 20 μg/mL in HeLa cells [121]. GO decreased the viability of A549 cells, while the same concentration and time of exposure increased the cell viability of CaCo2 colorectal carcinoma cells [122]. Another study reported that GO dramatically enhanced the differentiation of SH-SY5Y, accompanied by increasing neurite length and the expression of neuronal marker MAP2 at low concentrations but that GO suppressed the viability of SH-SY5Y cells at high doses (≥80 mg/mL) [123]. Functionalized coatings on GO, such as GO-PEG [124] and GO-chitosan [125], can profoundly attenuate the particles’ cytotoxicity by inhibiting the interactions between cells.

The toxicity of GFNs in vitro is summarized in Table 2. Data on the cytotoxicity of graphene nanomaterials are contrasting, and varying characteristics influence the results. The mechanisms and influencing factors of toxicity need to be elucidated in detail.

**Table 2:** Toxicity of GFNs in cell models

Graphene family nanomaterias	Physiochemial properties and Functionalization	Cells	Dose and time incubation	Effects	Reference
Pristine graphene	Thickness of 2–3 nm, size of 500–1000 nm	Murine RAW 264.7 macrophages	5, 10, 20, 40, 80 and 100 mg/mL, 48 h	Depleted of the mitochondrial membrane potential, increased ROS, triggered apoptosis	[83]
Pristine graphene	Thickness of 3–5 nm, size of 100–110 nm	Rat pheochromocytoma cells PC12 cells	10–100 μg/mL 1–48 h	Increased LDH release, ROS levels and caspase3 activation, induced apoptosis	[82]
Graphene oxide(GO)	Four different diameters (342–765 nm)	Human Erythrocytes Human skin fibroblasts CRL-2522	3.125-200 μg/mL 24 h	Hemolytic activity, ROS generation, LDH release, decreased cell viability	[106]
GO	Thickness of 0.9 nm lateral size: s-GO, 160 ± 90 nm; m-GO, 430 ± 300 nm; l-GO, 780 ± 410 nm	Human lung epithelial A549 cells	10, 25, 50, 100 and 200 μg/mL 24 h	Dose-dependent oxidative stress, cell viability decreased at high concentration	[119]
GO	Thickness of 1 nm, lateral dimension of 200–500 nm	Human lung fibroblast cells HLF cells	10–500 μg/mL 2–24 h	Oxidative stress induced, concentration-dependent cytotoxicity and genotoxicity	[148]
GO	Size distribution: 592 ± 10.9 nm in PBS, 1272 ± 56.2 nm in FBS	HeLa cells	0–80 μg/mL 24 h	Released LDH, increased MDA and ROS generation, decreased SOD, reduction of cell viability,	[120]
GO	smaller-sized GO: 50–350 nm intermediate-sized GO: 350–750 nm larger-sized GO: 750–1,300 nm	Macrophage cell J774A.1 THP-1 cells HEK293 cells MEL cells HUT102 cells	20 μg/mL 1-24 h	Size-dependent M1 induction of macrophages, pro-inflammatory responses	[94]
GO	thickness: < 2 nm, lateral size: 450 nm	Mouse CT26 colon carcinoma cell	50–100 μg/mL 18 h	Triggered autophagy, enhances cell death	[206]
Reduced graphene oxide (rGO)	Thickness of 11 ± 4 nm lateral size of 3.8 ± 0.4 μm	Human mesenchymal stem cells (hMSCs)	0.01–100 μg/mL 1–24 h	Induced DNA fragmentations and chromosomal aberrations	[118]
RGO	Thickness of 7 nm lateral size of 40 nm	human liver carcinoma cells (HepG2 cells)	1–200 mg/L 4–72 h	Dose-dependent DNA damage, oxidative stress, cytotoxicity	[31]
RGO	Lateral size of 100–1500 nm	U87 and U118 glioma cell lines	0–100 μg/mL 24 h	Reduction of cell proliferation and cell viability, induced apoptosis	[238]
Bacterially reduced graphene oxide (B-rGO)	Thickness of 4.23 nm average size of 3833 nm	MCF-7 cells	20–100 μg/mL 24–72 h	Increased ROS generation, released LDH, dose-dependent toxicity	[181]
Reduced graphene oxide Nanoribbons(rGONR)	Thickness of 1 nm, length of 10 μm, width of 50–200 nm,	hMSCs	0.01, 0.1, 1.0, 10, 100 μg/mL 96 h	Caused DNA fragmentations and chromosomal aberrations	[239]
Reduced graphene oxide sheets (rGOSs)	Thicknesses of ~1.2 nm, lateral sizes of ~2 μm	hMSCs	0.01, 0.1, 1.0, 10, 100 μg/mL 96 h	Caused slight cell membrane damage and cytotoxicity	[239]
Graphene-dextran (GO-DEX)	Thickness of 2.8 nm size of 50–100 nm	HeLa cells	10, 50,200 mg/L 24, 48, 72 h	GO-DEX remarkably reduced cell toxicity	[91]
GNP-COOH GNP-NH2	Thickness of GNP-COOH: 735.9 nm thickness of GNP-NH2: 945.5 nm	Human bronchial epithelial cells (BEAS-2B cells)	10, 50 mg/L 24 h	Caused single stranded DNA damage, genotoxicity and hypomethylation	[240]
PEG-DSPE (O-GNR-PEG-DSPE)	Width of 125–220 nm, lengths between of 500–2500 nm	HeLa cells NIH-3 T3 cells SKBR3 cells MCF7 cells	10–400 μg/mL 24–48 h	Dose-dependent and time-dependent decrease in cell viability	[138]
PEI-GO, PEG-GO, LA-PEG-GO	Thickness of 1–2 nm lateral width of 100–500 nm	Human lung fibroblast cells	1, 10, 50, 100 μg/ml 24 h	Caused concentration-dependent cytotoxicity and genotoxicity	[15]
PEG-GQD	Sizes of 3–5 nm	HeLa cells and A549 cells	10–160 μg/mL 24 h	No noticeable cytotoxicity	[176]
FBS-GO	Thickness of 4.0–18.0 nm	A549 cells	0–200 μg/mL 24 h	Cytotoxicity of GO was greatly mitigated at 10 % FBS	[166]

## Origins of GFNs toxicity

Reportedly, the characteristics of graphene, including its concentration, lateral dimension, surface structure, functional groups, purity and protein corona, strongly influence its toxicity in biological systems [2, 7, 104, 126–129].

### Concentration

Numerous results have shown that graphene materials cause dose-dependent toxicity in animals and cells, such as liver and kidney injury, lung granuloma formation, decreased cell viability and cell apoptosis [130–134]. In vivo studies, GO did not exhibit obvious toxicity in mice exposed to a low dose (0.1 mg) and middle dose (0.25 mg) but induced chronic toxicity at a high dose (0.4 mg). The high content of GO mainly deposited in the lungs, liver, spleen, and kidneys and was difficult to be cleaned by the kidneys via a single tail vein injection [135]. Intriguingly, increasing the dose resulted in a dramatic decrease in the hepatic uptake but an increase in the pulmonary uptake of s-GO by intravenous injection [31], because the high dose of GO potentially surpassed the uptake saturation or depleted the mass of plasma opsonins, which consequently suppressed the hepatic uptake. Moreover, an in vitro study reported that 20 μg/mL GO nanosheets exhibited no cytotoxicity in A549 within 2 h of incubation, but a higher concentration (85 μg/mL) decreased the cell viability to 50 % within 24 h [136, 137]. Lü et al. also demonstrated that GO had no obvious cytotoxicity at low concentrations for 96 h in a human neuroblastoma SH-SY5Y cell line, but the viability of cells sharply decreased to 20 % after treatment with 100 mg/mL GO for 96 h of incubation [123]. The results in HeLa cells, NIH-3 T3 cells, and breast cancer cells (SKBR3, MCF7) treated with graphene nanoribbons also showed a dose- (10–400 mg/ml) and time-dependent (12–48 h) decrease in cell viability [138]. Increasing concentrations of GO entered the lysosomes, mitochondria, endoplasm, and cell nucleus [119]. Several data indicated that rGO caused apoptosis-mediated cell death at a lower dose and early time point but that necrosis was prevalent with the increase in time/dose [110, 135].

### Lateral dimension

Nanoparticles with sizes <100 nm can enter the cell, <40 nm can enter nucleus, and smaller than <35 nm can cross the blood brain barrier [85]. One study showed that GO (588, 556, 148 nm) did not enter A549 cells and had no obvious cytotoxicity [112]. When the diameter of graphene is between 100 ~ 500 nm, the smallest size may cause the most severe toxicity, and when the diameter is below 40 nm, the smallest sizes may be the safest. For instance, rGO with a diameter of 11 ± 4 nm could enter into the nucleus of the hMSCs and cause chromosomal aberrations and DNA fragmentation at very low concentrations of 0.1 and 1.0 mg/mL in 1 h. However, rGO sheets with diameters of 3.8 ± 0.4 nm exhibited no notable genotoxicity in hMSCs even at a high dose of 100 mg/mL after 24 h [118].

In an in vivo study, s-GO (100–500 nm) preferentially accumulated in the liver, whereas l-GO (1–5 μm) was mainly located in the lungs because l-GO formed larger GO-protein complexes that were filtered out by the pulmonary capillary vessels after intravenously injection [31]. Given the relative lateral sizes (205.8 nm, 146.8 nm and 33.78 nm) of the three GO nanosheets at the same concentration, smaller GO experiences much greater uptake than larger GO in Hela cells [139]. The high uptake of s-GO changed in the microenvironment of cells and consequently induced the greatest viability loss and most serious oxidative stress among three sizes of GO samples [119]. As a result, one study delineated that GO size-dependently induced the M1 polarization of macrophages and pro-inflammatory responses in vitro and in vivo. Larger GO showed stronger adsorption onto the plasma membrane with less phagocytosis, eliciting robust interactions with TLRs and activating NF-κB pathways, compared to smaller GO sheets, which were more likely taken up by cells [94]. To further uncover the detailed mechanism underlying these effects, more studies are needed to illustrate the vital mechanism of the lateral size of graphene materials.

### Surface structure

**Fig. 2:**
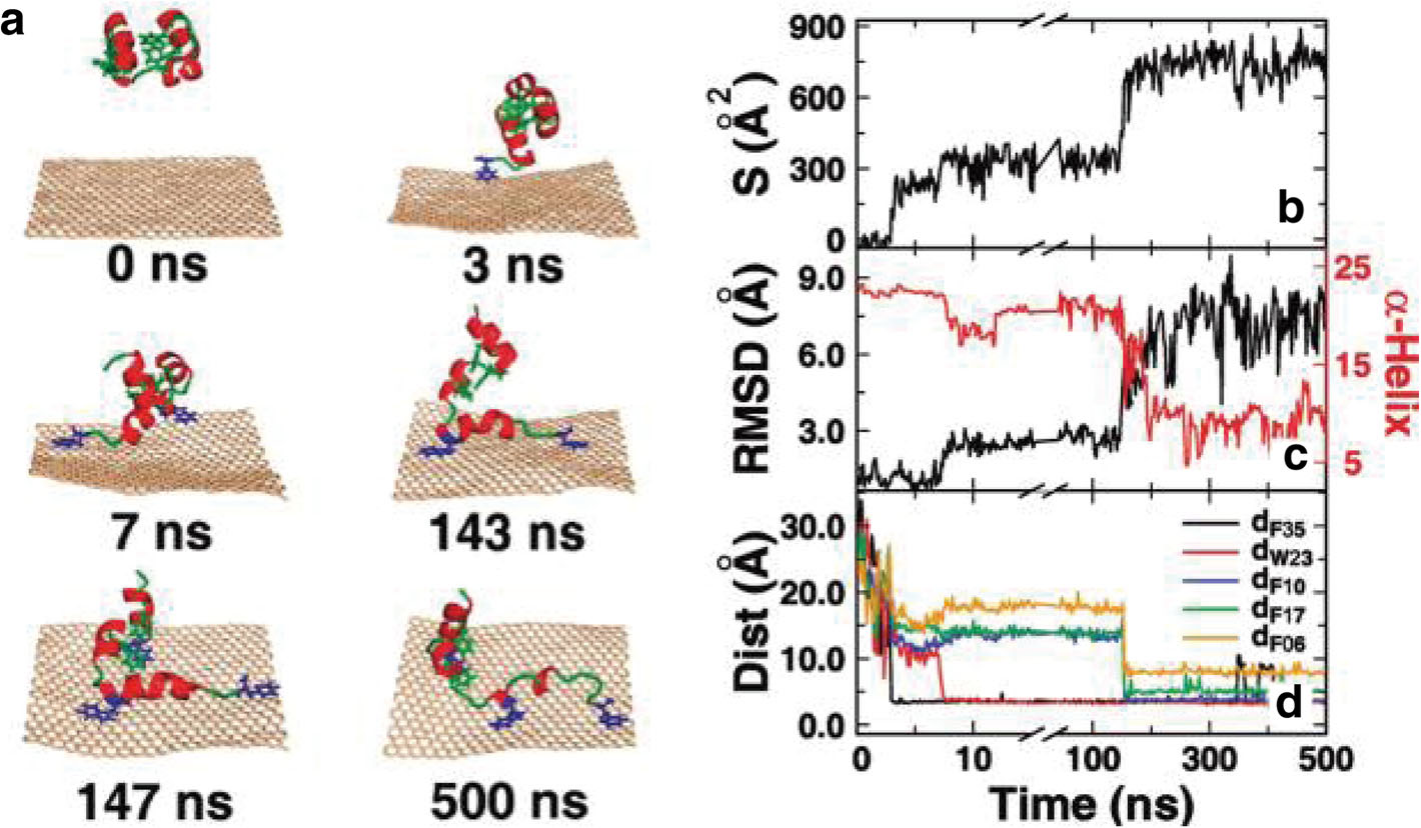
A representative trajectory of HP35 adsorbing onto the graphene. (**a**) Representative snapshots at various time points. The proteins are shown in cartoons with red helix and green loop, and the graphene is shown in wheat. The aromatic residues that form the π-π stacking interactions are shown in blue, others are shown in green. (**b**) The contacting surface area of HP35 with the graphene. (**c**) The RMSD of HP35 from its native structure and the number of residues in the α-helix structure. Here, the secondary structures are determined by the DSSP program. (**d**) The distance between the graphene and the aromatic residues, including F35, W23, F10, F17, and F06. To show the adsorbing process clearer, the χ-axis had been truncated and rescaled. [41] Copyright (2011), with permission from Journal of Physical of Chemistry

GFNs possess widely varying surface chemistries. For example, the pristine graphene surface is hydrophobic, GO surface is partially hydrophobic with carboxylate groups [140–142], and rGO has intermediate hydrophilicity [143]. GFNs were observed to disrupt the function and structure of cell membranes and proteins probably by exceptionally strong molecular interactions with cells [2, 91]. For instance, rGO bonded to cell membranes, stimulated receptors and activated mitochondrial pathways, inducing apoptosis [110, 111, 144]. Limited evidence showed that GO is smaller and less toxic than rGO because of the high oxygen content, smoother edges, and hydrophilic properties of the former species [104, 145, 146]. Because of the different surface oxidation states of GO and rGO, GO possessing distinct hydrophilicity might be internalized and taken up by HepG2 cells easily. On the contrary, rGO with evident hydrophobicity, could be adsorbed and aggregated at cell surfaces without (or with lower) uptake [110]. Due to strong π-π stacking interactions, graphene is highly capability of breaking many residues of the protein, particularly the aromatic ones, such as the villin headpiece (HP), F10, W23, and F35. The protein’s secondary and tertiary structures are largely lying on the graphene surface, disrupting the structure and function of the protein [41] (Fig. 2). In addition, GO can insert between the base pairs of double-stranded DNA and disturb the flow of genetic information at the molecular level, which might be one of the main causes of the mutagenic effect of GO [7, 112, 146, 147].

### Charge

A number of studies have highlighted the importance of the GO surface charge because of its ability to affect the internalization and uptake mechanism of cells [148–150]. GO internalization was negligible in non-phagocytes, which was likely due to the strong electrostatic repulsion between the negatively charged GO and the cell surface [34]. However, others have suggested that negatively charged nanoparticles can be internalized into non-phagocytic cells by binding to available cationic sites on the cell surface and be taken up by scavenger receptors [110, 146, 150]. GO/GS particles reportedly cause morphological changes and significant lysis, leading to high haemolysis in red blood cells (RBCs). RBC membrane disruption is probably attributed to the strong electrostatic interactions between the negatively charged oxygen groups on the GO/GS surface and positively charged phosphatidylcholine lipids on the RBC outer membrane [106].

### Functionalization

Studies confirmed that functionalization with PEG [52], PEGylated poly-L-lysine (PLL) [151], poly(ε-caprolactone) [152], polyvinyl alcohol [3], Pluronic [153], amine [98], carboxyl, and dextran [79] groups largely decreases the toxicity and improves the biocompatibility of graphene. In vivo results revealed that only mild chronic inflammation emerged after the subcutaneous injection of GO-Pluronic hydrogel and no noticeable short-term toxicity was tested after the intravenous injection of GO-DEX [79, 154]. PEGylated GS did not induce appreciable toxicity in mice exposed to 20 mg/kg for 3 months, as evaluated by blood biochemistry and histological examinations, and showed relatively low retention in the RES [52, 155]. Coating GO with chitosan almost eliminated the haemolytic activity in blood [39]. Moreover, the PEG coating effectively alleviated GO-induced acute tissue injuries; decreased GO aggregation and retention in the liver, lungs, and spleen; and promoted the clearance of GO [81], GO-DEX [79], and fluorinated graphene oxide (FGO) [156].

In vitro, several cell function assays showed clear evidence that the surface functionalization of pristine graphene or GO was critical for reducing the strong toxicity effects [91]. PEG-GO, PEI-GO and LA-PEG-GO damaged human lung fibroblast cells less than GO [148]. PEG-GO exhibited no cytotoxicity toward several cell cultures, such as glioblastoma cells (U87MG), breast cancer cells (MCF-7), human ovarian carcinoma cells (OVCAR-3), colon cancer cells (HCT-116), and lymphoblastoid cells (RAJI), at concentrations up to 100 μg/mL [119, 157, 158]. GQDs-PEG exhibited very low or no toxicity against lung and cervical cancer cells even at very high concentrations (200 μg/mL) [159]. However, as a non-biodegradable material with great potential for cellular internalization, further investigation is needed to assess the possible long-term adverse effects of functionalized graphene.

### Aggregations and sedimentation

Reportedly, nanomaterials have a propensity to form aggregates rather than individual units, particularly under physiological conditions. GS surfaces allowed fewer RBCs attach comparing to GO, and GS had the lower haemolytic activity for more aqueous aggregations formation. In contrast, the fast sedimentation and aggregate formation of GS greatly inhibited the nutrient availability of human skin fibroblast cells that were grown on the bottom of wells [106]. Therefore, the aggregations and sedimentation of graphene particles exert varying effects on different cells.

### Impurities

Nanomaterial purity is an important consideration because residual, contaminating metals may be responsible for the observed toxicity, rather than the nanomaterial itself, which has resulted in conflicting data on GFNs cytotoxicity [35, 160]. Traditionally prepared GO often contains high levels of Mn^2+^ and Fe^2+^, which are highly mutagenic to cells. The nonspecific release of these ions from traditionally prepared GO might lead to unusually high levels of cytotoxicity and DNA fracturing [39]. In particular, Peng et al. [161] produced high-purity GO containing only 0.025 ppm Mn^2+^ and 0.13 ppm Fe^2+^, and Hanene et al. [162] invented a new method to prepare high-purity, single-layer GO sheets with good aqueous dispersibility and colloidal stability. GO produced by these new methods did not induce significant cytotoxic responses (at exposure doses up to 100 μg/mL) in vitro, and no obvious inflammatory response or granuloma formation (exposure doses up to 50 μg/animal) were observed in vivo. Therefore, the purity of GFNs deserves attention and is a vital step towards the determination of GFNs involved in bioapplications.

### Protein corona effect

Because of the high free surface charge, nanomaterials can easily form “coronas” with proteins in biological systems [163, 164]. The protein corona is suggested to affect the circulation, distribution, clearance and toxicity of nanoparticles. Several papers reported that GO forms GO-protein coronas with adsorbed plasma proteins in serum and these GO-protein coronas play an important role in deciding the fate of the GO biokinetic behaviour in vivo. Such GO-protein coronas can regulate the adhesion of GO to endothelial and immune cells through both specific and nonspecific interactions [165]. Basically, immunoglobulin G and complement proteins in the protein corona help to reorganize nanoparticles in immune cells, causing the particles to be engulfed by the RES, and IgG-coated GO was taken up by either specific or nonspecific interactions with cell membrane receptors [31, 165]. However, another study found that GO could not adhere to mucosal epithelial cells directly in the intestinal tract after the filial mice drank an aqueous GO solution because abundant proteins in the milk had adsorbed on the surface of the GO and thus inhibited their direct interaction with the mucosal epithelial cells [53]. Protein corona mitigated the cytotoxicity of GO by limiting its physical interaction with the cell membrane and reducing the cellular morphological damage in HeLa, THP-1 and A549 cells [166–168]. The cytotoxic effect was largely reduced when GO was pre-coated with FBS and incubated with cells; nearly ∼ 90 % survival was observed with 100 μg/mL FBS-coated GO and 100 % survival with 20 μg/mL FBS-coated GO. Similar trends were observed for GO covered by BSA [166, 169]. Consistently, additional serum could neutralize the toxicity of pristine GO in J774.A1 cells at a dose of 4 μg/mL, which lead to a decrease in cell number of 52.5 % compared to untreated cells [89].

After reviewing many studies, it can be concluded that the toxicity of graphene is influenced by multiple factors. Those factors combined to largely change the toxicity of GFNs in many cases. Scientific studies often need the clear identification of cause and effect, which should keep only one factor different at a time, so that the effect of that single factor can be determined. But in some papers, several factors influencing GFNs toxicity were studied at the same time, which led to confused results.

## Possible toxicity mechanisms of GFNs

**Fig. 3:**
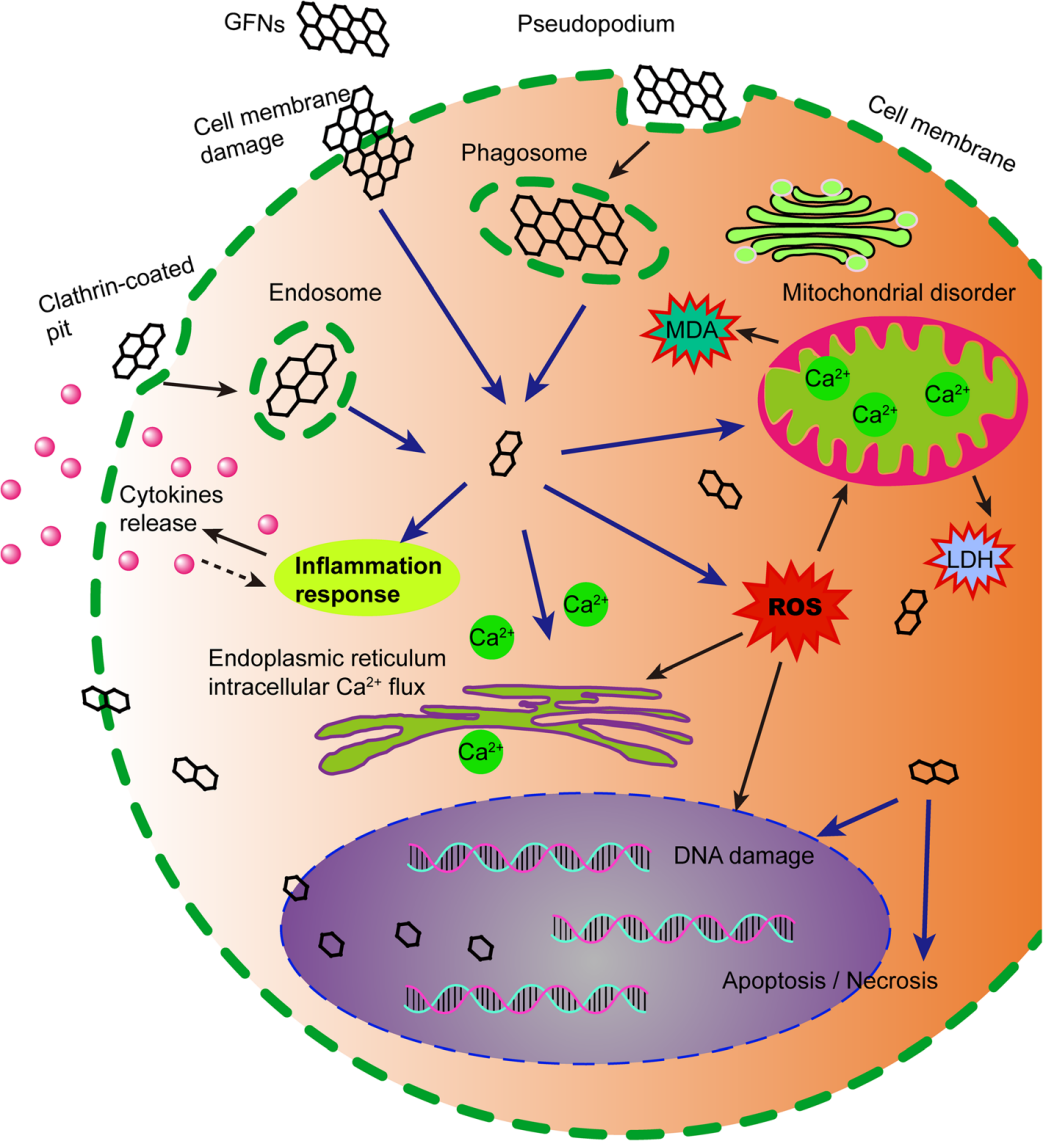
Schematic diagram showed the possible mechanisms of GFNs cytotoxicity. GFNs get into cells through different ways, which induce in ROS generation, LDH and MDA increase, and Ca^2+^ release. Subsequently, GFNs cause kinds of cell injury, for instance, cell membrane damage, inflammation, DNA damage, mitochondrial disorders, apoptosis or necrosis

Although some physicochemical properties and the toxicity of GFNs have been well studied by many scholars, the exact mechanisms underlying the toxicity of GFNs remain obscure. A schematic of the main mechanisms of GFNs cytotoxicity is illustrated in Fig. 3.

### Physical destruction

Graphene is a unique nanomaterial compared with other spherical or one-dimensional nanoparticles due to its two-dimensional structure with sp2-carbons. The physical interaction of graphene nanoparticles with cell membranes is one of the major causes of graphene cytotoxicity [7, 170, 171]. Graphene has high capability to bind with the α-helical structures of peptides because of its favourable surface curvature [172]. At concentration above 75 μg/mL, pristine graphene largely adhered to the surfaces of RAW 264.7 cells and resulted in abnormal stretching of the cell membrane [104]. The strong hydrophobic interactions of GFNs with the cell membrane lead to the morphological extension of F-actin filopodial and cytoskeletal dysfunction. Furthermore, the sharpened edges of GNS may act as ‘blades’, inserting and cutting through bacterial cell membranes [173]. Moreover, GO also damaged the outer membrane of *E. coli* bacteria directly, resulting in the release of intracellular components [173]. However, TEM imaging revealed that pre-coating GO with FBS eliminated the destruction of cell membranes [166].

### ROS production leading to oxidative stress

**Fig. 4:**
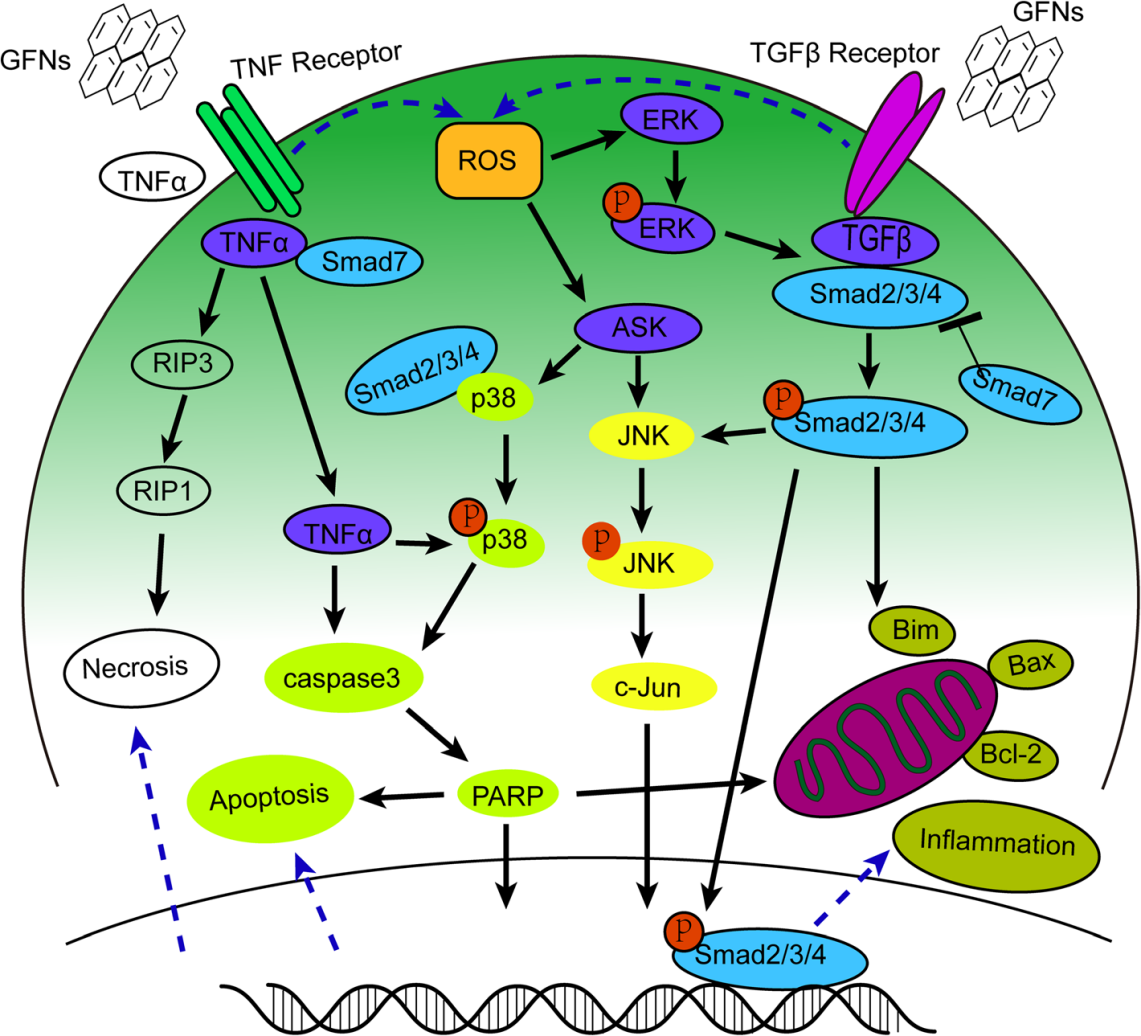
Schematic diagram of MAPKs, TGF-beta and TNF-α dependent pathways involved in GFNs toxicity. ROS was the main factors activating the MAPKs and TGF-beta signaling pathways to lead to the activation of Bim and Bax, triggering the cascade of caspases and JNK pathway. The activation of caspase 3 and RIP1 resulted in apoptosis and necrosis finally

Oxidative stress arises when increasing levels of ROS overwhelm the activity of antioxidant enzymes, including catalase, SOD, or glutathione peroxidase (GSH-PX) [174]. ROS act as second messengers in many intracellular signalling cascades and lead to cellular macromolecular damage, such as membrane lipid breakdown, DNA fragmentation, protein denaturation and mitochondrial dysfunction, which greatly influence cell metabolism and signalling [175–177]. The interactions of GO with cells can lead to excessive ROS generation, which is the first step in the mechanisms of carcinogenesis, ageing, and mutagenesis [83, 122]. Oxidative stress had a significant role in GO-induced acute lung injury [30], and the inflammatory responses caused by oxidative stress often emerged upon exposure to GFNs [133, 177, 178]. The activity of SOD and GSH-PX decreased after exposed to GO in a time- and dosage-dependent manner [82, 106, 119]. Similarly, oxidative stress was the key cause of apoptosis and DNA damage after HLF cells were exposed to GO [148]. Both the mitogen-activated protein kinase (MAPK) (JNK, ERK and p38) and TGF-beta-related signalling pathways were triggered by ROS generation in pristine graphene-treated cells, accompanied by the activation of Bim and Bax, which are two pro-apoptotic members of the Bcl-2 protein family. As a result, caspase-3 and its downstream effector proteins such as PARP were activated, and apoptosis was initiated [83, 179]. Detailed information regarding the MAPK-, TGF-β- and TNF-α-related signalling pathways, which induce inflammation, apoptosis and necrosis, are summarized in Fig. 4.

### Mitochondrial damage

Mitochondria are energy production centres involved in various signalling pathways in cells and are also a key point of apoptotic regulation [83]. After exposure to GO and carboxyl graphene (GXYG), the mitochondrial membrane was depolarized, and the amount of mitochondria decreased in HepG2 cells [180]. Exposure to GFNs resulted in significantly increased coupled and uncoupled mitochondrial oxygen consumption, dissipation of the mitochondrial membrane potential, and eventual triggering of apoptosis by activating the mitochondrial pathway [181]. For instance, GO increased the activity of mitochondrial electron transport complexes I/III and the supply of electrons to site I/II of the electron transport chain, accelerating the generation of ROS during mitochondrial respiration in MHS cells [99]. The formation of •OH mediated by GO and the cytochrome-c/H_2_O_2_ electron-transfer system could enhance oxidative and thermal stress to impair the mitochondrial respiration system and eventually result in dramatic toxicity [151]. Additionally, the oxygen moieties on GO might accept electrons from cellular redox proteins, supporting the redox cycling of cytochrome c and electron transport proteins, and cytochromes MtrA, MtrB, and MtrC/OmcA might be involved in transferring electrons to GO [182]. Therefore, except for the plasma membrane damage and oxidative stress induction, GFNs can cause apoptosis and/or cell necrosis by direct influencing cell mitochondrial activity [183, 184].

### DNA damage

Due to its small size, high surface area and surface charge, GO may possess significant genotoxic properties and cause severe DNA damage, for example, chromosomal fragmentation, DNA strand breakages, point mutations, and oxidative DNA adducts and alterations [87, 122, 185, 186]. Mutagenesis was observed in mice after intravenous injection of GO at a dose of 20 mg/kg compared with cyclophosphamide (50 mg/kg), a classic mutagen [112]. Even if GO cannot enter into the nucleus of a cell, it may still interact with DNA during mitosis when the nuclear membrane breaks down, which increases the opportunity for DNA aberrations [87, 147, 187, 188]. The π stacking interaction between the graphene carbon rings and the hydrophobic DNA base pairs can make a DNA segment ‘stand up’ or ‘lay on’ the surface of graphene with its helical axis perpendicular or parallel, respectively. The intermolecular forces severely deform the end base pairs of DNA, which potentially increases the genotoxicity [189]. GO may also induce chromosomal fragmentation, DNA adducts and point mutations by promoting oxidative stress or triggering inflammation through the activation of intracellular signalling pathways such as MAPK, TGF-β and NF-κB [110, 112, 146]. Graphene and rGO can also elevate the expression of p53, Rad51, and MOGG1-1, which reflect chromosomal damage, and decrease the expression of CDK2 and CDK4 by arresting the cell cycle transition from the G1 to the S phase in various cell lines [112]. DNA damage can not only initiate cancer development but also possibly threaten the health of the next generation if the mutagenic potential of GO arises in reproductive cells, which impacts fertility and the health of offspring [112, 190].

### Inflammatory response

**Fig. 5:**
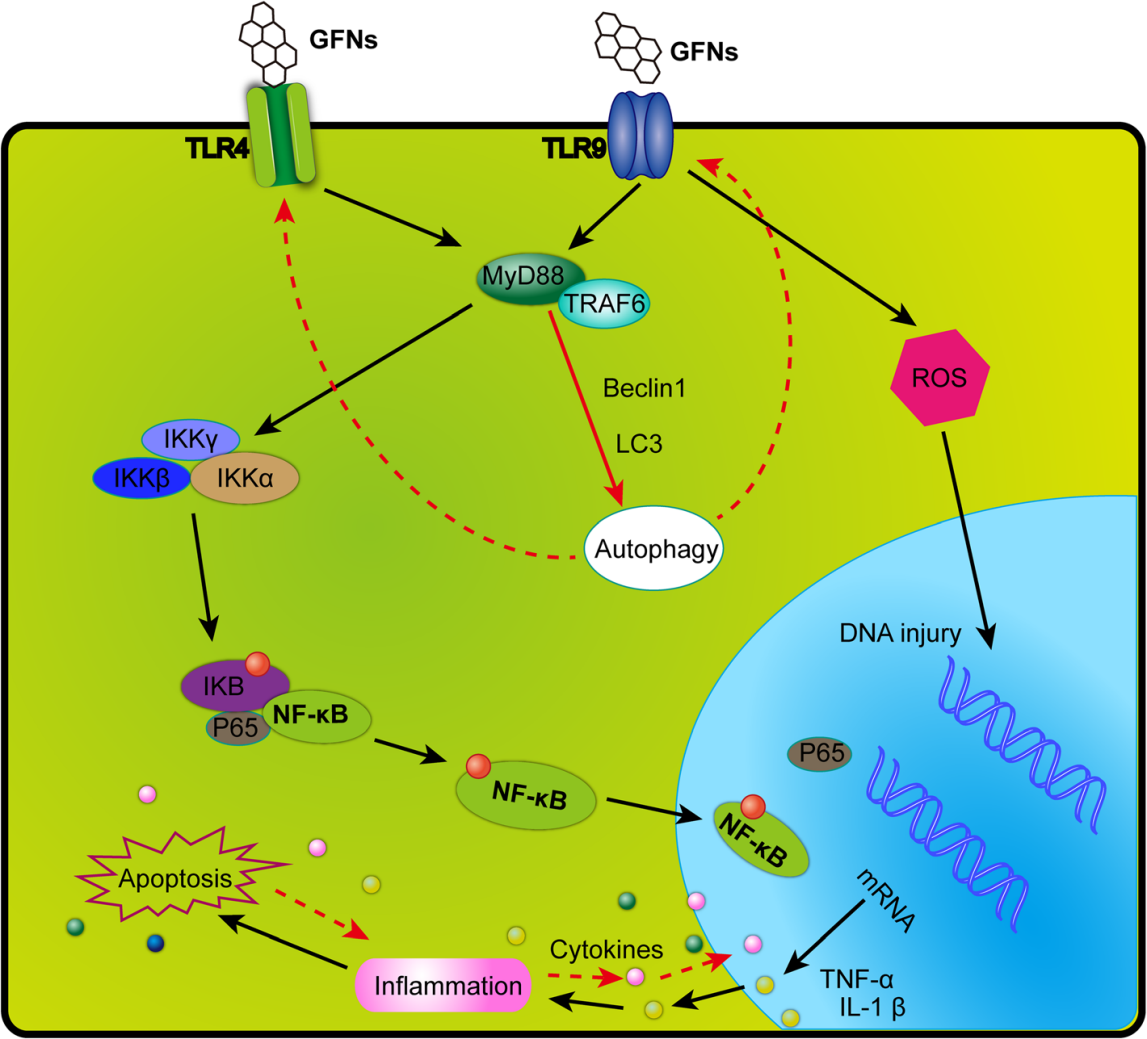
A schematic diagram elucidating signalling pathway of TLR4 and TLR9 responsible for GFNs-induced cytotoxicity. GFNs can be recognized by TLRs, thus activate IKK and IκB by a MyD88-dependent mechanism, resulting in the release of NF-κB subunits and initiating the translocation into the nucleus. Thus, pro-inflammatory factors were transcribed and secreted out of nucleus, modulating the immune responses initiating programmed autophagy, apoptosis and necrosis

GFNs can cause a significant inflammatory response including inflammatory cell infiltration, pulmonary edema and granuloma formation at high doses via intratracheally instillation or intravenous administration [30, 49]. Platelets are the important components in clot formation to attack pathogens and particulate matter during the inflammatory response, and GO could directly activate platelet-rich thrombi formation to occlude lung vessels after intravenous injection [98, 191]. A strong inflammatory response was induced by subcutaneously injection with GO for 21 days, along with the secretion of key cytokines, including IL-6, IL-12, TNF-α, MCP-1, and IFN-g [34, 192]. GFNs can trigger an inflammatory response and tissue injury by releasing cytokines and chemokines that lead to the recruitment of circulating monocytes and stimulating the secretion of Th1/Th2 cytokines and chemokines [124, 193]. Additionally, pristine graphene [193] and rGO [110] evoke an inflammatory response by binding to toll-like receptors (TLRs) and activating the NF-κB signalling pathway in cells. The NF-κB signalling cascade is triggered by TLRs and pro-inflammatory cytokines such as IL-1 and TNF-α. Upon activation, NF-κB shifts from the cytoplasm to the nucleus, facilitating the binding of degrading IκB and acting as a transcription factor to synthesize numerous pro-inflammatory cytokines [194]. A schematic of the signalling pathway of TLR4 and TLR9 activated by GFNs is shown in Fig. 5.

### Apoptosis

Apoptosis is defined as the self-destruction of a cell regulated by genes through complicated programmes [83, 195]. GO and rGO caused apoptosis and inflammation in mice lungs after inhalation [99], and GFNs also had pro-apoptotic effects in cells [111, 113, 124, 196]. Additionally, graphene and GO physically damaged cell membranes [166], increased the permeabilization of the outer mitochondrial membrane and changed the mitochondrial membrane potential; the increased ROS triggered the MAPK and TGF-β signalling pathways and activated caspase-3 via mitochondrial-dependent apoptotic cascades, prompting the execution of apoptosis [83, 99]. Similarly, rGO caused apoptosis at a low dose and an early time point, triggered by the death-receptor and canonical mitochondrial pathway [110]. Another study showed three different apoptosis pathways by GFNs: GO led to ROS-dependent apoptosis through direct interaction with protein receptors and subsequent activation of the B-cell lymphoma-2 (Bcl-2) pathway; GO-COOH transmitted a passive apoptosis signal to nuclear DNA by binding to protein receptors and activating a ROS-independent pathway; However, GO-PEI severely damaged the membranes of T lymphocytes to trigger apoptosis [105, 197].

### Autophagy

Autophagy is the process of self-degradation of cellular components and recently recognized as non-apoptotic cell death [198–200]. Autophagy activation requires autophagosome formation containing Beclin 1, multiple autophagy-related proteins (ATG), microtubule-associated protein light chain 3 (LC3) and p62 [201]. Autophagosome accumulation is associated with exposure to various nanoparticles [202–205], and autophagy can remove extracellular organisms and destruct the organisms in the cytosol [206]. GO and GQDs was shown to induce autophagosome accumulation and the conversion of LC3-I to LC3-II; inhibit the degradation of the autophagic substrate p62 protein [207, 208]. Furthermore, GO can simultaneously trigger TLR4 and TLR9 responses in macrophages [34, 192] and colon cancer cells CT26 [206]. The autophagy pathway is linked to phagocytosis by TLR signalling in macrophages [206, 209].

### Necrosis

Necrosis is an alternate form of cell death induced by inflammatory responses or cellular injury. The exposure of cells to pristine graphene causes apoptosis and necrosis at high doses (50 mg/mL) [83]. Reportedly, LDH leakage and the opening of the mitochondrial permeability transition pore, induced by elevated level of cytoplasmic Ca^2+^, lead to apoptosis/necrosis [210]. GO treatment was revealed to induce macrophagic necrosis by activating TLR4 signalling and subsequently partly triggering autocrine TNF-α production [93]. GO combined with CDDP (GO/CDDP) triggered necrosis by decreasing RIP1 and increasing RIP3 proteins, accompanied with the release of high mobility group B1 (HMGB1) into the cytosol from the nucleus and out of CT26 cells [205, 211, 212].

### Epigenetic changes

Epigenetics involve DNA methylation, genomic imprinting, maternal effects, gene silencing, and RNA editing [213–215]. DNA methylation, which is one of the best-studied epigenetic modifications, includes phosphorylation, ubiquitination, and ATP-ribosylation and can lead to chromatin remodelling [197, 216, 217]. A recently paper reported that SL-GO/FL-GO exposure resulted in global DNA hypermethylation through upregulating DNMT3B and MBD1 genes; GNP treatment caused hypomethylation by decreasing the expression of DNMT3B and MBD1 genes [216]. GO could activate the miRNA-360 regulation pathway to suppress the DNA damage-apoptosis signalling cascade by affecting the component of CEP-1 [218]. Taken together, these data suggest that GFNs could cause subtle changes in gene expression programming by modulating epigenetic changes. However, studies of GFNs-induced epigenetic changes are few, and the epigenetic mechanism caused by GFNs exposure is not fully understood.

To conclude, many studies have discussed representative mechanisms of GFNs toxicity involving four signalling pathways: TLRs, TGF-β, TNF-α and MAPKs. These four signalling pathways are correlative and cross-modulatory, making the inflammatory response, autophagy, apoptosis and other mechanisms independent and yet connected to each other. Additionally, oxidative stress appears to play the most important role in activating these signalling pathways. It has been reported that there are intersections of apoptosis, autophagy and necrosis in the studies of other nanomaterials toxicity, they inhibit or promote mutually in some conditions. However, the signalling pathways of GFNs toxicity investigated in papers to date are only a small part of an intricate web, and the network of signalling pathways needs to be explored in detail in the future.

## Data gaps and future studies

Currently, the literature is insufficient to draw conclusions about the potential hazards of GFNs. Two opposite opinions have begun to emerge: some researchers suggested that graphene materials are biocompatible in a number of studies focused on biomedical applications [119, 154, 162, 219], and other studies reported adverse biological responses and cytotoxicity [32, 118, 135, 138, 192]. These inconsistent results might have been caused by several factors, including the different research groups, various cellular or animal models, and varying physicochemical characterizations of GFNs. When GFNs are explored for in vivo applications in the human body or some other biomedical applications, biocompatibility must be considered, and more detailed and accurate studies of GFNs toxicity are needed.

First, detailed physicochemical characterization is imperative in all future studies of GFNs toxicity. In the experiments, feature descriptions of GFNs should include their size, morphology, surface area, charge, surface modifications, purity, and agglomeration [88, 141, 148, 162]. Because these physicochemical factors largely influence the toxicity and biocompatibility of GFNs, single-factor experimental designs and the exclusion of other interfering factors should be considered. Details of the fabrication process should also be provided because the formed oxidative debris could largely alter the surface structure of graphene and GO during functionalization [151]. Importantly, a single, universal method needs to be established in graphene technology, which will allow for better comparison of data from different studies or different laboratories.

Second, different observational criteria, parameters and selection of experimental methods might induce large inter-laboratory variations [220, 221]. For example, the MTT assay always fails to accurately predict graphene toxicity because the spontaneous reduction results in a false positive signal. Therefore, appropriate alternative assessments should be utilized, such as the water-soluble tetrazolium salt reagent (WST-8), ROS assay, and trypan blue exclusion test [106, 222]. Additionally, the comet assay often shows higher levels of DNA damage than the micronucleus assay because the former measures the repairable injury and the latter measures the gene damage that remains after cell division [159, 223]. Therefore, caution is required in choosing the most appropriate assay to evaluate the toxicity of graphene materials to avoid false-positive results.

Third, the selection of cell lines is of vital importance because cancer cell lines tend to be sensitive or resistant depending upon their genetic background. The same graphene nanoparticles can cause different reactions depending on their various cells origins. Suitable cell lines with good stability must be used to avoid false positive or negative results. Primary cells derived from humans or animals can better simulate the health conditions of humans. A large amount of primary cells have been utilized to test the toxicity of other nanomaterials [224–228], but the culturing of primary cells is extremely rare in the experiments with GFNs to date [210, 229]. Various cell experiments combined with primary cells should be performed to comprehensively evaluate the physicochemical properties and toxicity of GFNs.

Fourth, the administration route of GFNs plays a very important role in toxicity studies, and different delivery methods will result in different toxicological reactions [32, 53]. Thus, the route and period of exposure should be carefully chosen according to the aim of the study. Nasal drug delivery is often used to study the neurotoxicity of nanomaterials [230, 231], but this administration method has rarely been applied in the testing of GFNs toxicity. Toxicological studies of GFNs in the nervous system are rare, and the mechanism is unclear and needs to be studied further in the future. Recent toxicokinetic studies involving the absorption, distribution, metabolism, accumulation, and excretion of GFNs through different exposure routes have yielded some results but are far from sufficient to clarify the internal complex mechanisms. For instance, further studies are needed to understand the specific molecular mechanisms of GFNs passing through the physiological barriers and the amount of accumulation or the excretion period of GFNs in tissues. In addition, given the increased exposure of humans to GFNs, the assessment of systemic toxicity in the human body is indispensable in future studies.

Fifth, another important issue requiring attention is the long-term fate of GFNs after entering the body or being taken up by cells. Most recent studies have consisted of short-term toxicity assessments [89, 232], and long-term toxic injury has not received much attention since the widespread application of GFNs in 2008. Moreover, a functionalized graphene surface can improve its biocompatibility, but the long-term stability of the surface coatings should be considered [233]. If the surface coatings eventually break down, their toxicity may be significantly different from the short-term exposure results. Extended studies are needed to determine if longer treatment times influence the nanotoxic potential of GFNs.

Sixth, more specific signalling pathways in the mechanism of GFNs toxicity need to be discovered and elucidated. Currently, several typical toxicity mechanisms of GFNs have been illustrated and widely accepted, such as oxidative stress, apoptosis, and autophagy. However, these mechanisms have only been described in general terms, and the specific signalling pathways within these mechanisms need to be investigated in detail. The signalling pathways involved in the toxicity of other nanomaterials may also be relevant to the study of GFNs. Therefore, more signalling pathways should be detected in future research. For instance, nano-epigenetics has been considered in numerous studies of nanomaterials, which is also helpful in assessing the limited toxicity and side effects of GFNs. Recent studies have shown that GFNs could cause epigenetic and genomic changes that might stimulate physical toxicity and carcinogenicity [234]. GFNs have high surface areas, smooth continuous surfaces and bio-persistence, similar to the properties of tumorigenic solid-state implants. It is unknown whether GFNs have the potential to induce foreign body sarcomas, and definitive studies of tumour potentialities or risks of graphene should therefore be conducted as soon as possible.

## Conclusions

In the past few years, GFNs have been widely utilized in a wide range of technological and biomedical fields. Currently, most experiments have focused on the toxicity of GFNs in the lungs and livers. Therefore, studies of brain injury or neurotoxicity deserve more attention in the future. Many experiments have shown that GFNs have toxic side effects in many biological applications, but the in-depth study of toxicity mechanisms is urgently needed. In addition, contrasting results regarding the toxicity of GFNs need to be addressed by effective experimental methods and systematic studies. This review provides an overview of the toxicity of GFNs by summarizing the toxicokinetics, toxicity mechanisms and influencing factors and aimed to provide information to facilitate thorough research on the in vitro and in vivo haemo- and biocompatibility of GFNs in the future. This review will help address safety concerns before the clinical and therapeutic applications of GFNs, which will be important for further development of GFNs in biological applications.

## Abbreviations

AMs: Alveolar macrophages
BBB: Blood-brain barrier
BEB: Blood-epididymis barriers
BTB: Blood-testis barrier
CR: Complement receptor
FcgR: Fcg receptor
FLG: Few-layer graphene
GFNs: Graphene family nanomaterials
GNS: Graphene nanosheets
GO: Graphene oxide
GO-COOH: Carboxylated graphene oxide
GO-DEX: GO-dextran
GO-MB: GO-molecular beacon
GO-NH2: Aminated GO
GO-PAA: Poly(acrylic acid)-functionalized GO
GO-PAM: Poly(acrylamide)-functionalized GO
GO-PEG: PEGylated GO derivatives
GO-PEI: GO-polyethylenimine
GQDs: Graphene quantum dots
GSH-PX: Glutathione peroxidase
GXVG: Carboxyl graphene
LDH: Lactate and dehydrogenase
MALDI: Matrix-assisted laser desorption/ionization
MAPKs: Mitogen-activated protein kinase
MDA: Malondialdehyde
MØ: Macrophage
MR: Mannose receptor
MSI: Mass spectrometry imaging
PC12 cells: Rat pheochromocytoma cells
PCGO: Protein-coated graphene oxide nanoparticles
PrGO: PEGylated reduced graphene oxide
RES: Reticuloendothelial system
rGO: Reduced graphene oxide
ROS: Reactive oxygen species
SOD: Superoxide dismutase
TLRs: Toll-like receptor
